# Virulence factor-related gut microbiota genes and immunoglobulin A levels as novel markers for machine learning-based classification of autism spectrum disorder

**DOI:** 10.1016/j.csbj.2020.12.012

**Published:** 2020-12-29

**Authors:** Mingbang Wang, Ceymi Doenyas, Jing Wan, Shujuan Zeng, Chunquan Cai, Jiaxiu Zhou, Yanqing Liu, Zhaoqing Yin, Wenhao Zhou

**Affiliations:** a Shanghai Key Laboratory of Birth Defects, Division of Neonatology, Children’s Hospital of Fudan University, National Center for Children's Health, Shanghai 201102, China; b Research Center for Translational Medicine, Koç University, Istanbul, Turkey; c Division of Neonatology, The People's Hospital of Dehong Autonomous Prefecture, Mangshi, Yunnan 678400, China; d Division of Neonatology, Longgang District Central Hospital of Shenzhen, Guangdong 518116, China; e Division of Neurosurgery, Tianjin Children’s Hospital, Tianjin 300134, China; f Division of Psychology, Shenzhen Children’s Hospital, Shenzhen, Guangdong, China; g Yanqing Liu,Guangzhou Medical University, Guangzhou, Guangdong 510623, China; h State Key Laboratory of Medical Neurobiology and MOE Frontiers Center for Brain Science, Institutes of Brain Science, Fudan University, Shanghai, 201102, China

**Keywords:** ASD, autism spectrum disorder, IgA, immunoglobulin A, LPS, lipopolysaccharide, TD, typical development, VFDB, virulence factor database, VFGM, virulence factor-related gut microbiota, Autism spectrum disorder, Classification, Early diagnosis, Genetics, Gut microbiota, Immunoglobulin A, Machine learning, Metagenome, Virulence factor

## Abstract

Autism spectrum disorder (ASD) is a neurodevelopmental condition for which early identification and intervention is crucial for optimum prognosis. Our previous work showed gut Immunoglobulin A (IgA) to be significantly elevated in the gut lumen of children with ASD compared to typically developing (TD) children. Gut microbiota variations have been reported in ASD, yet not much is known about virulence factor-related gut microbiota (VFGM) genes. Upon determining the VFGM genes distinguishing ASD from TD, this study is the first to utilize VFGM genes and IgA levels for a machine learning-based classification of ASD. Sequence comparisons were performed of metagenome datasets from children with ASD (*n* = 43) and TD children (*n* = 31) against genes in the virulence factor database. VFGM gene composition was associated with ASD phenotype. VFGM gene diversity was higher in children with ASD and positively correlated with IgA content. As Group B streptococcus (GBS) genes account for the highest proportion of 24 different VFGMs between ASD and TD and positively correlate with gut IgA, GBS genes were used in combination with IgA and VFGMs diversity to distinguish ASD from TD. Given that VFGM diversity, increases in IgA, and ASD-enriched VFGM genes were independent of sex and gastrointestinal symptoms, a classification method utilizing them will not pertain only to a specific subgroup of ASD. By introducing the classification value of VFGM genes and considering that VFs can be isolated in pregnant women and newborns, these findings provide a novel machine learning-based early risk identification method for ASD.

## Graphical abstract

**Figure:**
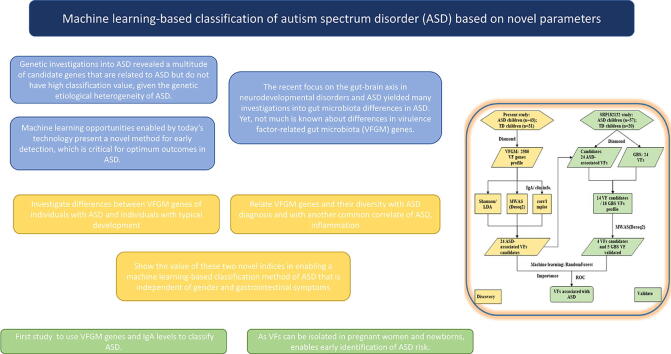

## 1 Introduction

Autism spectrum disorder (ASD) is a neurodevelopmental condition that affects 1 in 54 (1.9%) of 8-year-old children in the US [1], and 0.29% of 6 to 12-year-old children in China [2]. For this condition with increasingly rising prevalence and no known single cause or cure, comprehensive molecular diagnostic testing continues to reveal previously unidentified copy number and single nucleotide variants, reasserting the genetic heterogeneity of ASD and highlighting the difficulty of a molecular diagnosis for ASD [3]. Gut microbiota has been linked to many central nervous system disorders, including neuropsychiatric disorders such as ASD [4]. Several studies suggest that changes in the microbiota may contribute to the symptoms of ASD [5], and fecal microbiota transfer therapy has been shown to significantly improve autism symptoms [6], suggesting that gut microbiota may contribute to the pathogenesis of ASD. Yet, limited studies to date explored the genetics of the gut microbiota in ASD, especially those related to virulence factors. Virulence factors (VFs) are molecules produced by bacteria, viruses, fungi, and protozoa that enable a microorganism to establish itself on or within a host of a particular species and enhance its potential to cause disease [7].

Our previous work showed gut Immunoglobulin A (IgA) content to be significantly elevated in the gut lumen of children with ASD compared to typically developing (TD) children [8]. IgA is an antibody that plays a crucial role in the immune function of mucous membranes, as it regulates antibody responses against commensal species [9]. Therefore, gut IgA is instrumental in controlling the composition of commensal microbiota [10]. In return, the IgA content and diversity in gut is strongly affected by the gut microbiota [11], where gut IgA content refers to IgA concentration in the gut.

Machine learning, which refers to the study of computers learning patters in empirical data [12], has started to be used to identify markers to distinguish children with ASD from TD children. Maenner et al. developed a machine learning model based on words and phrases to predict the status of ASD cases, which is used to evaluate whether children on surveillance sites meet ASD surveillance criteria [13]. In the meantime, machine learning methods based on electroencephalography data have also achieved good performance in distinguishing ASD children from TD children, with an accuracy of > 85% in different studies [14], [15], [16]. Machine learning methods have also been widely used in metagenomics research to discover the composition of disease-related intestinal flora [17], [18]. In our previous study, we found that the tryptophan pathway is associated with major depression based on machine learning methods [17]. Recently, machine learning was used with gut microbiota to classify ASD with an AUC value of 0.768 [18], yet no studies looked at the genetics of the gut microbiota for such classification via machine learning. Machine learning techniques are increasingly being used to understand relationships between microbial pathogens and mammalian hosts [19] and are being preferred to other modeling methods in genetic investigations where knowledge about underlying mechanisms remain insufficient [20], as is also the case with microbial alterations in ASD. Additionally, viruses in the gut have been implicated in pathogenesis of certain conditions as they are suggested to induce alterations in DNA of the bowel wall and influence immune homeostasis [21]. Combining these utilization avenues of machine learning with implications about gut viruses in pathogenesis and their relations with the immune system, the present study aimed to predict virulence factor-related gut microbiota (VFGM) genes and investigate the connection of these VFGM genes with ASD and gut IgA, and thereupon test a machine learning-based classification system for ASD using VFGM genes and IgA. Since not much is known about the genetics of VFGM in ASD, establishing its connections with other reported physiological alterations in ASD such as immunological aberrations via IgA measurements is important to understand how these different components relate with each other and play a role in the etiology of ASD.

## 2 Materials & methods

### 2.1 Participants

The recruitment of children with ASD and TD has been described in detail in previous studies [8], [24]. In short, the diagnosis of ASD followed DSM-5 and the ASD symptoms were further evaluated by Childhood Autism Rating Scale (CARS) [25]. The inclusion criteria for ASD group included being aged between 2 and 8 years of age and having a diagnosis of ASD according to the criteria of DSM-5; the inclusion criteria for TD group was being age-matched typically developing children. The exclusion criteria for ASD and TD groups were having a diagnosis of mental conditions other than ASD such as attention-deficit hyperactivity disorder or obsessive compulsive disorder, and having used antibiotics within the past month. For all enrolled children, signed informed consent was obtained from the parents. The protocol of this study was in accordance with the Declaration of Helsinki and was approved by the Ethics Committee of Shenzhen Children's Hospital.

### 2.2 Clinical phenotype evaluation and gut IgA content detection

Collection of clinical information, the evaluation of gastrointestinal (GI) symptoms using Rome IV criteria, collection of participant stool samples and IgA content in gut lumen, and shotgun metagenome sequencing and analysis were performed as detailed in previous studies [8], [26]. Feature GI was used as binary variable for further analyses. For clinical information collection, the Rome IV criteria for functional gastrointestinal disorders was used for evaluating GI symptoms. IgA content in gut lumen was measured from participants’ stool samples by enzyme-linked immunosorbent assay, as in our previous studies [8], [26], [27]. Fecal supernatant was added to an ELISA plate pre-coated with goat anti-human IgA, IgG, IgM antibody (Jackson ImmunoResearch, West Grove, USA), the plate was incubated at 37 °C for 1 h, then horseradish peroxidase labeled goat anti-human IgA (Jackson ImmunoResearch) was added, and finally absorbance at 450 nm was detected. Feature IgA was used as continuous variable for further analyses.

### 2.3 Shotgun metagenome sequencing

Following previously used procedures [24], to collect stool samples, participants discharged the feces in the designated manure collection basin, and participants’ parents prepared the medical disposable stool sampling tubes. They wore gloves, collected feces, collected the middle section, and avoided inadvertent pollution. If any contamination was found, they were instructed to resample. After completion, the samples were quickly frozen and stored in a refrigerator at −20 °C. On the present or the next day, the researchers took the sample with an incubator containing dry ice and stored it in a −80 °C refrigerator for later use.

Shotgun Metagenome sequencing and taxonomic annotation of shotgun metagenomics dataset were performed, following previous studies [24], [28]. DNA from stool samples were extracted using StoolGen DNA kit (CWBiotech Co., Beijing, China), and the libraries were constructed with a TruSeq DNA Sample Preparation kit (Illumina, San Diego, CA, USA), and libraries were sequenced using an Illumina Hiseq4000 sequencer (Illumina, San Diego, CA, USA), reads with empty adapter or low quality were filtered out, and all reads were aligned to the human reference genome Hg19 to filter out reads with possible human contamination.

### 2.4 VFGM gene prediction and metagenomic analysis in the present study

To obtain the VFGM gene composition, we first downloaded the most recent version of VFDB [29] as a reference VFs database, and then the reference database was built using Diamond’s [30] makedb, finally, the Diamond’s blastx method was used to get VFGM gene abundance profile, where the parameters were “*--evalue 1e-5 --threads 4 --*max*-target-seqs 1 --outfmt 6*″.

The impact of each clinical index on VFGM composition was evaluated based on Permutational Multivariate Analysis of Variance Using Distance Matrices (PERMANOVA) method via the use of VEGAN package (version 2.0–9) in R (Version 3.6.3). Sample diversity was measured using the formula: normalized Shannon diversity index = $\sum_{i}$-log($p_{i}$)*$p_{i}/log\left(n\right)$, where *n* is the number of reads blasted to the VFDB and *p_i_* is the frequency of each VFGM gene *i*. The Wilcoxon rank-sum test was used to compare the diversity differences between groups, and *p* values <0.05 were considered statistically significant. To determine whether the diversity indices were associated with IgA content, we performed a correlation analysis using cor.test () function in R, and linear regression analysis using lm () function in R.

To determine whether VFGM gene composition can be used to distinguish children with ASD from TD children, Linear Discriminant Analysis (LDA) was also performed using ggord package (version 1.1.5, http://fawda123.github.io/ggord/). Differences in VFGM genes between ASD and TD were determined using Deseq2 package (version 1.4.5), false discovery rate (FDR)-corrected *p* < 0.0001 was considered significant.

In order to visualize the significant differences VFGM genes between ASD and TD children, and evaluate the correlation between the VFGM genes and clinical indicators, the violinplot function and lmplot function of the seaborn package (version 0.10.1) were used to draw the violinplot and lmplot, respectively; statannot (version 0.2.3) was used for the analysis of differences between groups, and the selected test method was Mann-Whitney; the stats function of the scipy package (version 1.5.0) was used for linear regression analyses.

### 2.5 Candidate VFs and GBS genes validation with the SRP182132 study

Group B streptococcus, or *Streptococcus agalactiae* (GBS) genes accounted for the highest proportion of 24 different VFGMs. As gut GBS genes abundance and gut IgA level, the potential biomarkers of ASD we found [8], [26], were positively correlated, we further evaluated if the different VFGMs, together with GBS VF genes, were potential ASD markers. For this purpose, the published metagenome data by Kovtun et al. [31] (referred to as the SRP182132 study) was first downloaded, and then the abundance of 24 different VFGMs and GBS VF genes were predicted. In short, the 24 different VFGMs from VFDB and all GBS VF protein sequences from the Victors pathogen database [32] were downloaded as reference, and then the reference database was built using Diamond’s [30] makedb. Finally, the Diamond’s blastx method was used to get VFs gene abundance profile, where the parameters were “*--evalue 1e-5 --threads 4 --*max*-target-seqs 1 --outfmt 6*″. As mentioned before, the differences in VF genes between ASD and TD were determined using the Deseq2 package (version 1.4.5) and *p* < 0.05 was considered significant for the data of the SRP182132 study.

### 2.6 Machine learning

First, the train_test_split function from the model_selection module of the sklearn package (version 0.23.1) in Python (version 3.7.6) was used to split the present study and SRP182132 study into training and test data sets, the set parameters are: test_size = 0.4, random_state = 2020; Then, the RandomForestClassifier from the ensemble module of the sklearn package was imported as RandomForest model, and the parameters of the RandomForest model were set as: random_state = 0, n_estimators = 100, oob_score = True, n_jobs = -1; Then, the RandomForest model was fitted based on the training data set, the feature_importances_ function of the RandomForest model was used to rank the importance of features, and barh function from pyplot module of the matplotlib package (version 3.2.2) in Python was used to visualize important features. Finally, the roc_curve and auc function from metrics module of the sklearn package were used for ROC analysis and AUC score evaluation on the test samples respectively, and plot module of the matplotlib package was used for visualization. In order to get the 95% confidence interval of the AUC score on test samples, the cross_val_score function from model_selection module of the sklearn package was used to perform cross-validation analysis, the parameter is cv = 5, and the 95% confidence interval is computed as: scores_cv.mean() ± scores_cv.std() * 1.96.

## 3 Results

### 3.1 VFGM gene composition associated with ASD phenotype

**Fig. 1:**
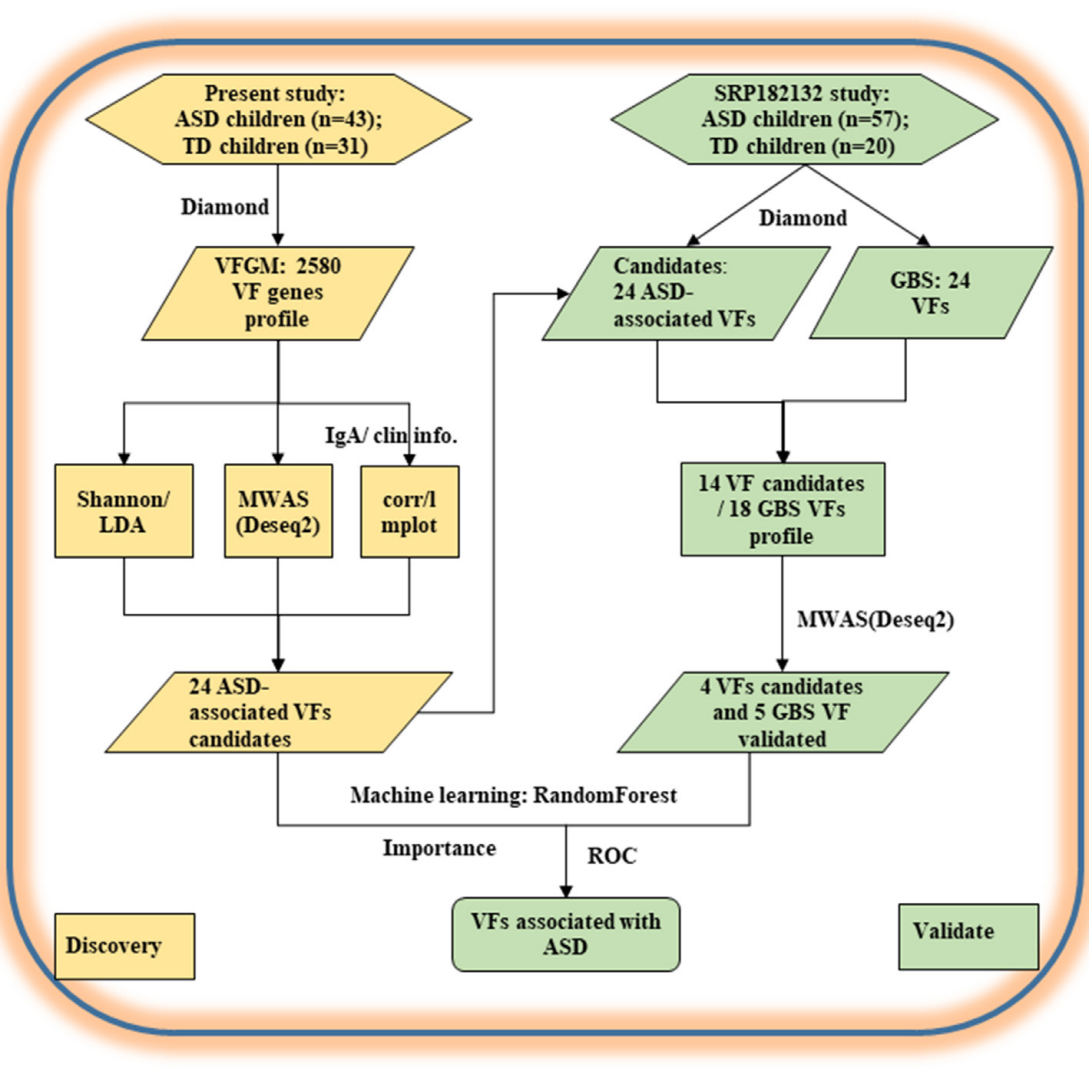
Study design flowchart. ASD, Autism Spectrum Disorder; TD, Typical Development; VFDB, virulence factor database; VFGM, virulence factor-related gut microbiota; IgA, immunoglobulin A.

A total of 43 children with ASD (7 females, ranged 2–6 years old), together with 31 TD children (14 females, range 2–5 years old) participated in this study. The study design was shown in Fig. 1.

A total of 2580 VFGM genes have been predicted using the VFDB database. To determine whether the clinical phenotype was significantly associated with VFGM gene composition, we performed PERMANOVA analysis, and the results (Supplementary Table 1) show that the ASD phenotype (*p* = 0.0001), gut IgA content (*p* = 0.0294), and prolonged labor (*p* = 0.0057) significantly associated with VFGM gene composition. Other clinical variables, such as age, sex, GI symptoms, family history of neurological diseases and other clinical indices, were not associated with VFGM gene composition. Therefore, we next focused on evaluating the relationships between IgA content, ASD phenotype, and VFGM gene composition.

### 3.2 VFGM gene diversity is positively correlated with IgA content, and VFGM gene composition distinguishes between children with ASD and TD children

**Fig. 2:**
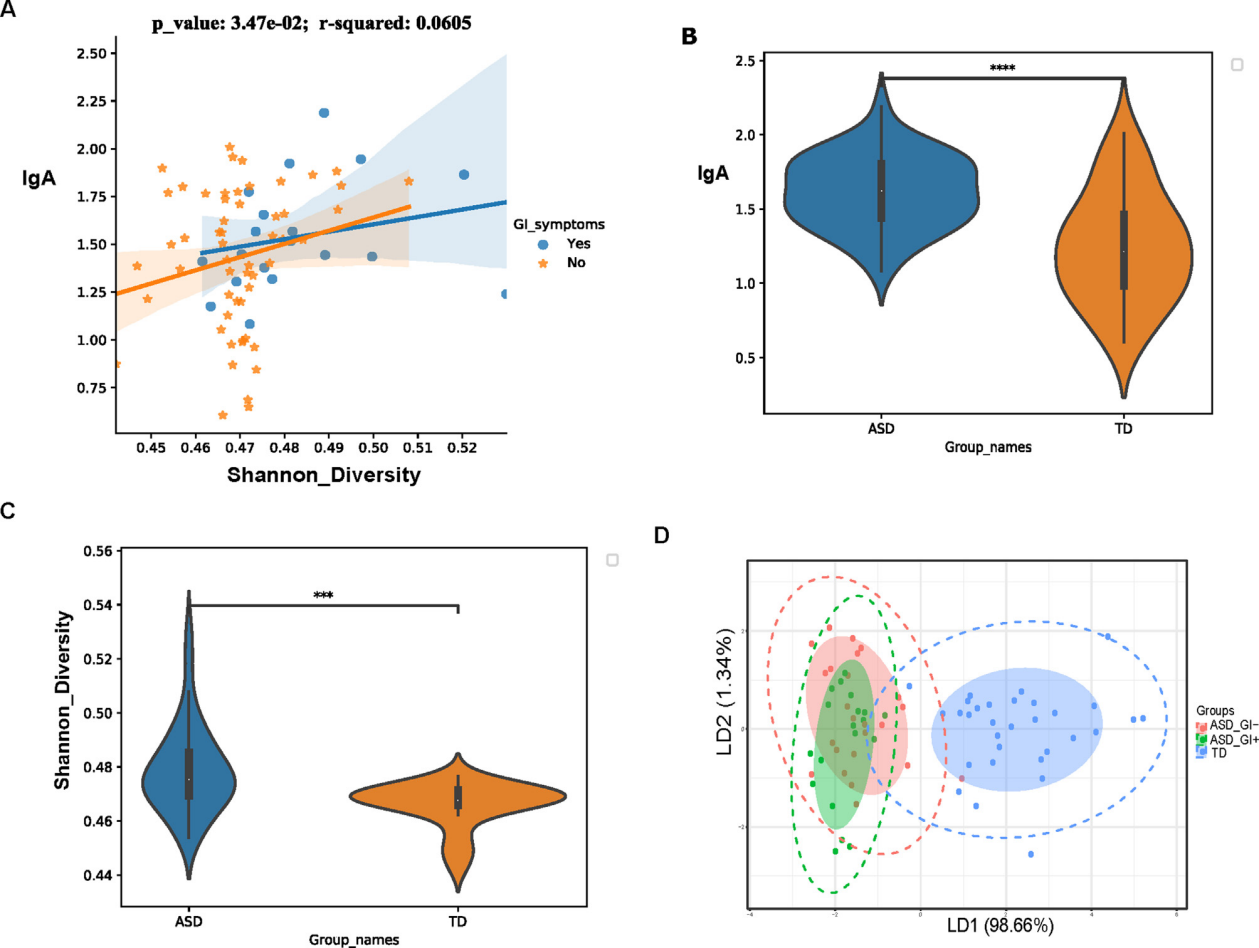
Comparison of VFGM gene between ASD and TD. a, Correlation and linear regression analysis of Shannon diversity index for VGFM and IgA content; b, Comparison of gut IgA level between ASD and TD children; c. compared VFGM gene diversity between the ASD and TD groups; d, LDA analysis showing that gene composition clearly distinguishes between children with ASD and TD children; Wilcoxon rank sum test, ***p < 0.001, **p < 0.01, *p < 0.05.

Since IgA content was significantly associated with VFGM gene composition, we computed the Shannon diversity of VFGM genes for each sample and analyzed their correlations with IgA content. The results showed that VFGM gene Shannon diversity positively correlated with IgA content (Fig. 2a, *p* = 0.0347). We also found that in children with ASD, the gut IgA level was significantly higher than that of TD children (Fig. 2b and Supplementary Fig. 1a-b, P < 0.05, Wilcoxon rank sum test). To further assess whether VFGM gene Shannon diversity was a potential biomarker for ASD children, we first compared the difference in VFGM gene Shannon diversity between ASD children and TD children, and found that the difference was significant (Fig. 2c). We also found that GI symptoms and sex were not factors that lead to the difference in VFGM diversity between children with ASD and TD. (Supplementary Fig. 1c-d). To evaluate whether VFGM gene composition could be a potential biomarker for ASD, we performed LDA analysis and found that ASD cases could be clearly separated from TD cases based on VFGM gene composition (Fig. 2d).

### 3.3 Several VFGM genes are differentially enriched in children with ASD

We next determined whether any of the identified VFGM genes were specifically enriched or depleted in children with ASD. Using DESeq2 [26], we identified a total of 24 ASD patient-specific VFGM gene markers (FDR-corrected *p* < 0.0001; Supplementary Table 2). Seventeen VFGM genes were significantly enriched in children with ASD, including two type III secretion system (T3SS) genes, *OspC4* and *espA*, from species *Shigella flexneri 2a str. 301* and *Escherichia coli O157:H7 str. EDL933*, respectively; two type IV secretion system genes, *lpg2552* and *legK2*, from species *Legionella pneumophila* subsp. *pneumophila str. Philadelphia 1*; two cytolysin genes, *cylI* and *cylR1*, from species *Enterococcus faecalis str. MMH594*; four lipopolysaccharide (LPS) genes, *acpXL*, *kfiC*, *wlaN*, and *Cj1137c*, the latter two of which are from species *Campylobacter jejuni* subsp. *jejuni NCTC 11168*; and seven capsule and surface antigen-related genes, *csbD*, *hasA*, *capC*, *cpsJ*, *cpsH*, *cpsM*, *and casO*, the latter four of which are from species *Streptococcus agalactiae 2603 V/R*.

Seven differentially enriched VFGM genes were significantly depleted in children with ASD compared with TD children, including *afaC-VII*, an afimbrial adhesin AFA-VII (AI014) gene from species *Escherichia coli str. 239 KH 89*; *wcbE*, a glycosyltransferase from species *Burkholderia pseudomallei K96243*; *dotB*, a Dot/Icm type IV secretion system ATPase from species *Legionella pneumophila* subsp. *pneumophila str. Philadelphia 1*; *flgF*, a flagellar basal body rod protein from species *Burkholderia pseudomallei K96243*; *mtrE*, a multidrug efflux pump channel protein gene from species *Neisseria meningitidis MC58*; *pvdM*, a dipeptidase precursor gene from species *Pseudomonas aeruginosa PAO1*; and *tcpI*, a toxin co-regulated pilus biosynthesis protein I gene from species *Vibrio cholerae O1 biovar El Tor str. N16961*.

### 3.4 VFGM genes are related with ASD

**Fig. 3:**
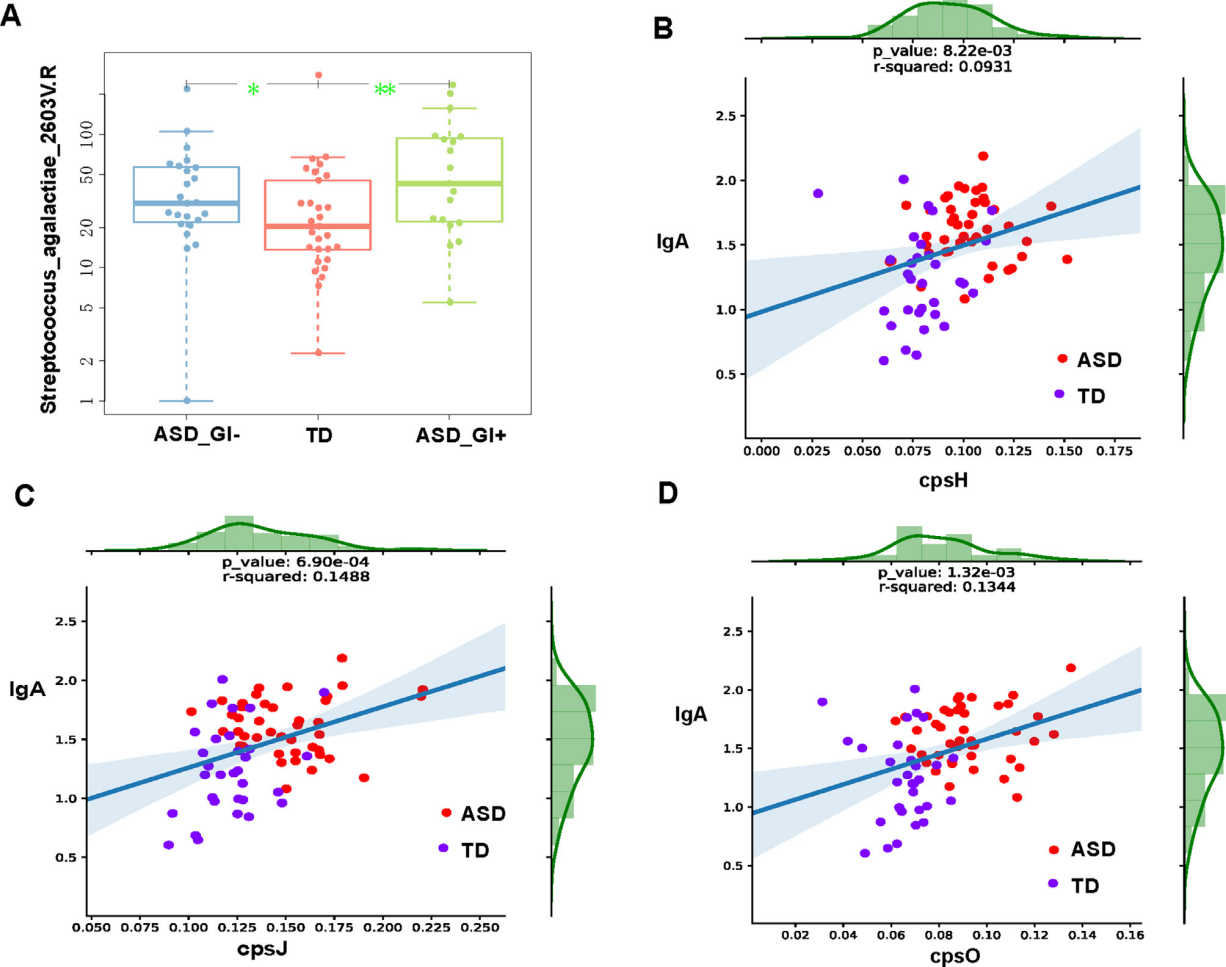
Association of VFGM genes with ASD. a, increased abundance of *Streptococcus agalactiae 2603 V/R* in gut of children with ASD with/without GI symptoms (ASD_GI+/ASD_GI-) compared with TD children, *, P < 0.05, **, P < 0.01, Wilcoxon Rank Sum test; b-d, increased abundance of *Streptococcus agalactiae 2603 V/R* gene cpsH (c), cpsJ (d), and cpsO (e) are associated with rise of gut IgA level.

To further evaluate the 24 ASD-related VFGM genes, we correlated them with Shannon diversity and IgA content. We found that three of nine ASD-enriched VFGM genes, *cpsH*, *cpsJ,* and *cpsO*, were from *Streptococcus agalactiae 2603 V/R*, and the abundance of *Streptococcus agalactiae 2603 V/R* was increased in gut of ASD children with/without GI symptoms compared with that of TD children, (Fig. 3a), and the significant increase in gut IgA levels in children with ASD was independent of the GI symptoms. It is worth noting that we found that the gut abundance of three GBS VFs, *cpsH*, *cpsJ* and *cpsO* to be positively correlated with gut IgA levels (Fig. 3b-d).

### 3.5 Machine learning prediction based on VF

**Fig. 4:**
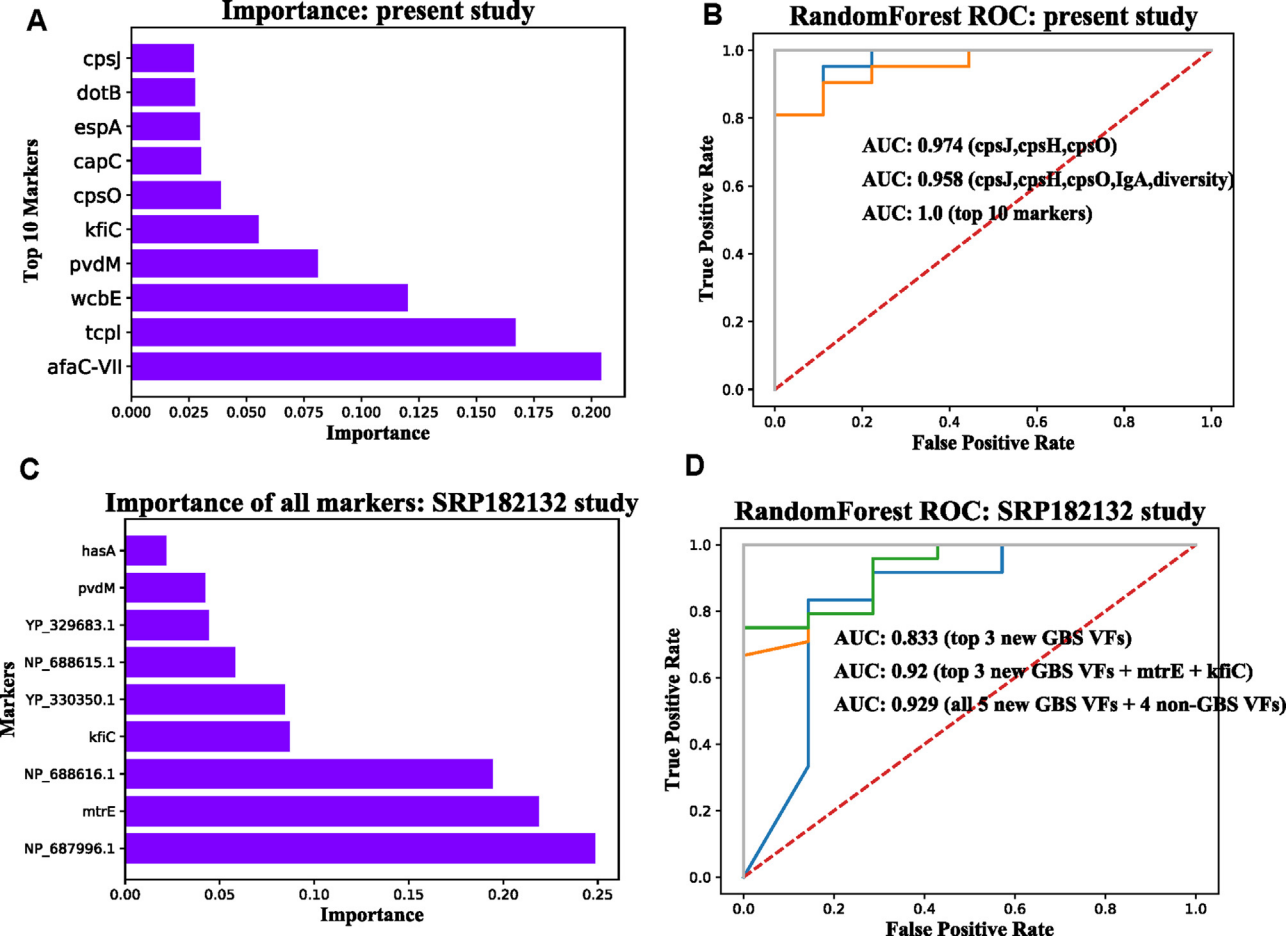
GBS genes as potential biomarkers for ASD diagnosis. RandomForest and testing samples were used, a, the rank of top ten importance of VFGM genes, gut VFGMs diversity index, gut IgA level, and clinical indices for ASD diagnosis in the present study; b, three GBS genes (*cpsJ*, *cpsH*, *cpsO*) had a AUC score of 0.974 in the present study, three GBS genes (*cpsJ*, *cpsH*, *cpsO*) combined with IgA and gut VFGMs diversity index had an AUC score of 0.974 in the present study; c, the rank of top ten importance of new GBS genes and clinical indicators for ASD diagnosis in the SRP182132 study; d, top 3 new GBS genes had an AUC score of 0.833 in the SRP182132 study, top 3 new GBS genes combined with other non-GBS VF genes (*mtrE* and *kfiC*) had an AUC score of 0.92 in the data of the SRP182132 study, all 5 new GBS genes combined with other non-GBS VF genes (*mtrE*, *kfiC*, *pvdM* and *hasA*) had an AUC score of 0.929 in the data of the SRP182132 study.

We used the machine learning method RandomForest to evaluate variables, including differential VFGMs, gut VFGMs diversity index, gut IgA level, and clinical indices for ASD diagnosis. The importance of variables, which is an indicator in RandomForest that marks the contribution of a variable to distinguish cases with ASD from control, is ranked in Fig. 4a and Supplementary Fig. 3a. Finally, we also used the machine learning method Randomforest to construct binary classifiers to distinguish the ASD and TD groups based on VF markers. The performance of binary classifiers was evaluated with Receiver Operating Characteristic (ROC) curves and Area Under ROC Curve (AUC) score, where AUC score ranged from 0.5 to 1 and a higher AUC score indicates better performance for classifiers. It is worth noting that the three GBS genes (*cpsH*, *cpsJ*, *cpsO*) had an AUC score of 0.974 (95% CI: 0.83 ± 0.21), and three GBS genes (*cpsJ*, *cpsH*, *cpsO*) combined with IgA and gut VFGMs diversity index had an AUC score of 0.958 (95% CI: 0.97 ± 0.13), top 10 VFs had an AUC score of 1.0 (95% CI: 0.97 ± 0.13) (Fig. 4b).

Given that we have previously found GBS genes to account for the highest proportion of 24 different VFGMs, and that gut GBS gene abundance was positively correlated with gut IgA level, we further evaluated the correlation between VFs of GBS and ASD. We first downloaded the publicly published ASD gut metagenomic dataset (SRP182132), where the abundance of gut GBS VF genes, together with 24 different VFGMs, were predicted. A total of nine VFs were significantly decreased in gut of ASD compared with controls in the SRP182132 study, including five new GBS genes, namely *YP_329683.1*, *NP_688616.1*, NP_687996.1, *NP_688615.1*, *YP_330350.1* and five non-GBS VF genes identified in the present study, namely *kfiC*, *pvdM*, *mtrE* and *hasA*. Then, we used the machine learning method Randomforest method to analyze the importance of these markers (Fig. 4c). The results showed that top 3 new GBS genes had an AUC score of 0.833 (95% CI: 0.74 ± 0.33) in the SRP182132 study, top 3 new GBS genes combined with two non-GBS VF genes (*mtrE* and *kfiC*) had an AUC score of 0.92 (95% CI: 0.84 ± 0.36) in the data of the SRP182132 study, and that all 5 new GBS genes combined with 4 non-GBS VF genes (mtrE, kfiC, pvdM and hasA) had an AUC score of 0.929 (95% CI: 0.87 ± 0.24) in the data of the SRP182132 study (Fig. 4d).

## 4 Discussion

Autism spectrum disorder (ASD) is a condition that eludes a single etiology. The social nature of the impairments it creates both delays diagnosis and makes adaptation to mainstream communal contexts challenging. Early diagnosis and intervention are key in achieving the best outcomes and improvement in behaviors and communication. Machine learning approaches hold the potential to be harnessed to obtain such early diagnosis. To date, machine learning has been used to classify cases that fall into an ASD diagnosis for prevalence surveillances [13] and to distinguish ASD cases based on gut microbiota genera [18]. In this study, we utilized two novel parameters, virulence factor-related gut microbiota (VFGM) genes and IgA, for a machine learning-based classification of ASD.

First, we found that VFGM gene diversity was higher in children with ASD than in TD children, and was positively correlated with IgA content in children with ASD. These findings implicated VFGM and IgA as potential biomarkers to distinguish between children with ASD and TD children. Using the machine learning method on VFGM gene diversity, VF markers, and IgA was able to distinguish children with ASD and TD.

Among the 17 ASD-enriched VFGM genes, 7 genes were associated with IgA and Shannon diversity, including 4 VFGM genes, *capC*, *cpsH*, *cpsJ*, and *cpsO* encoded surface protein as potential antigen, three of which (*cpsH*, *cpsJ*, and *cpsO*) were derived from *Streptococcus agalactiae 2603 V/R*. Previous studies have shown that group B *streptococcus* (GBS) can induce maternal immune activation in pregnant mice and leads to autistic-like phenotype in offsprings [33], [34], indicating that *streptococcus* may be potential pathogenic factors for ASD. Our results agreed with previous studies and suggested that *cpsH*, *cpsJ*, and *cpsO* derived from *Streptococcus agalactiae 2603 V/R* were potential biomarkers of ASD, and subsequent in-depth studies can help determine whether they are involved in the etiology of ASD. Three LPS genes, *kfiC*, *Cj1137c,* and *wlaN*, were significantly enriched in children with ASD and positively correlated with gut IgA and VFGM gene diversity. Our results are consistent with previous studies showing that prenatal exposure to LPS induced maternal immune activation (MIA) and lead to autistic-like behaviors in rats [35], [36], [37]. *lpg2552*, a T3SS gene used by pathogenic bacteria to inject virulence proteins into host immune cells and regulate the immune response [36], and *cylI*, a cytolysin protein encoding gene associated with quorum-sensing autoinduction [37], were also significantly enriched in children with ASD and positively correlated with gut IgA and VFGM gene diversity. Previous studies have shown that cytolysin itself may also stimulate NF-ĸB-mediated pro-inflammatory responses [38], and that potential pathogen *Enterococcus faecalis* has been found to sense target cells via cytolysin expression [39]. These are consistent with our observations that both *cylI* and *Enterococcus faecalis* were enriched in gut of ASD children compared with TD children.

In terms of machine learning-based classification, the three GBS genes *cpsH*, *cpsJ*, *cpsO* had an AUC score of 0.974, and when combined with IgA and gut VFGMs diversity index, revealed an AUC score of 0.958, which is better than the AUC value of 0.768 obtained when gut microbiome was used for a machine-learning based ASD classification recently [18]. As GBS is one of the most common bacteria that infect pregnant women and GBS-induced maternal immune-activated rats often show behaviors similar to ASD [33], [34], our findings implicate these genes as potential biomarkers to enable detection of ASD risk from as early as birth.

This study and its findings are important on four levels: First, previous genetic investigations into ASD have revealed a multitude of candidate genes that are related to ASD but do not have high classification value due to their multitude and heterogeneity across cases. Second, the recent interest in the gut-brain axis in neurodevelopmental disorders and especially ASD yielded many investigations into gut microflora differences in ASD, but not much is known about the differences in VFGM genes of individuals with ASD. Third, the relationship of these genes with another common correlate of the condition, inflammation, and with an ASD diagnosis is investigated for the first time. Fourth, the value of these two novel indices in enabling a machine learning-based classification method of ASD is evidenced.

VFGM gene diversity is thought to reflect the diversity of pathogenic bacteria in the gut; thus, the higher VFGM gene diversity in children with ASD may indicate a greater risk for pathogenic bacterial invasion compared with TD children. The “gut–brain axis” hypothesis proposes a role for the gut in ASD pathogenesis [5], and children with ASD often show gastrointestinal problems [40]. The recent finding that fecal microbiota transfer can improve the core symptoms of children with ASD [6] suggest an important, and possibly pathogenic, role for the gut microbiota in ASD. Our finding that VFGM gene diversity was higher in children with ASD compared with TD children supports the possibility that pathogenic gut microbes may be associated with the development of ASD.

Two forms of IgA are present in the gut lumen; natural (polyspecific) and antigen-specific. IgA content and diversity are associated with multiple factors, including subject age, gut microbiota composition, and T cell abundance [41]. IgA-responsive bacteria can be enriched and identified using the recently developed method of IgA-seq, a flow cytometry-based bacterial cell sorting and 16S sequencing method [42]. Recent studies found that bacteria enriched by IgA-seq were directly involved in the development of inflammation, inflammatory bowel disease (IBD) [44], and spondyloarthritis [45], Oral W27 IgA, an IgA that specifically binds to non-beneficial bacteria, is a potential treatment for IBS [46]. Therefore, measuring the gut IgA content is important because the IgA content may indirectly reflect the composition and diversity of disease-associated gut microbiota. We found that VFGM gene diversity was positively associated with IgA content, and that both VFGM gene diversity and IgA content were higher in children with ASD (with or without GI symptoms) than in TD children. Thus, we speculate that children with ASD may carry higher levels of IgA-responsive bacteria, referring to bacteria that may introduce host IgA, some of which may be tied to the development of ASD. Furthermore, although the gut IgA content showed a strong correlation with VFGM diversity in the total sample, this correlation was revealed to only be present in the ASD group (Fig. 2a), suggesting that IgA content may be a response to the diversity of VFGM in ASD children, but not in TD children. Studies have also found that IgA-responsive bacteria may be associated with IBD etiology [44], [46].

Of the seven ASD-depleted VFGM genes, six were associated with Shannon diversity and IgA content. The lower VFGM gene abundance in children with ASD may mean that they have less exposure to potentially pathogenic bacteria. Our results are consistent with a previous study, which found that less exposure to potential pathogens *Listeria monocytogenes*, *bacillus* species, and *corynebacterium* species was related to a risk of autoimmune diseases such as asthma in children [47]. Considering that the presence of autoimmune diseases is an important risk factor for ASD [48], and that the occurrence of autoimmune diseases is closely related to gut microbiota imbalance [49], our observation of VFGM gene imbalance may imply abnormal immune function in gut of individuals with ASD, which makes sense in relation to the differential IgA levels observed in this group. Our study on virulence factors extends such previous findings to show how they relate with other immune agents and how they can distinguish the gut microsystem of individuals with ASD from that of TD individuals.

We recently investigated potential common mechanisms of action for inflammation and gut microbiota on the neural basis of ASD, and proposed them to be their effects on neurodevelopment, ASD-susceptibility genes, and intestinal and blood–brain barrier integrity [50]. Our findings extend this theoretical model by showing how virulence factor-related gut microbiota genes can also be important, alongside ASD-susceptibility genes, in regulating the gut microbiota and related inflammatory status of the gut, and thus may have consequences for ASD neurology. The recent finding that ASD microbiota induces alternative splicing of ASD-implicated genes in the brain [51] suggests a connection between the virulence factor-related gut microbial genes and the genetic expression in the brain, which may connect the gut and the brain within the genetic sphere. Another recent finding shows that oral probiotic administration to pregnant MIA mice prevented the emergence of ASD-like behaviors normally seen in MIA offspring and the MIA-induced increases in pro-inflammatory cytokines interleukin 6 (IL-6) and IL-17a, suggesting that probiotics may induce their effects on the gut microbial system in ASD via their anti-inflammatory effects [52]. Thus, our findings involving VFGM genes and IgA levels add to the recently emerging modelings of the gut-immune-brain system for understanding and treating ASD, and contribute the dimension of genetics to this axis.

Finally, since not all individuals with ASD have abnormal EEG findings, previous reports of machine learning classification methods based on EEG may only be applicable to certain subgroups of individuals with ASD. However, as VFGM gene diversity, IgA increase, and ASD-enriched VFGM genes were found in this study to be independent of sex and GI symptoms, basing machine learning on these parameters may make it more broadly applicable to larger ASD populations.

### 4.1 Limitations and future directions

To our knowledge, this is the first study to use a machine learning-based classification of ASD based on VFGM genes and IgA levels. However, our study has some limitations, such as the sex was not perfectly matched between ASD and TD children as the male-to-female ratio in ASD children reached 4:1 or more, which reflects the ASD prevalence in the general population. Additionally, although fungi are also increased in the gut of children with ASD [51], the reference VFDB database used here contains only bacterial pathogens.

In the future, we will systematically validate the differentially enriched VFGM genes in a large-scale study with a larger sample. We will continue our investigations to verify that these VFGM genes are ASD-specific markers, and determine whether VFGM gene diversity and VFGM gene-specific IgA could be used as reliable biomarkers before or shortly after birth, since some different VFs detected in ASD came from the pathogen GBS that can be isolated in pregnant women and newborns. Finally, future investigations into whether the increase in IgA content is due to VFGM genes may inform ASD models by providing another puzzle piece for the mystery of autism etiology.

## 5 Conclusion

The two important findings of this study are: (1) VFGM gene diversity positively correlated with IgA content and both VFGM gene diversity and IgA content were higher in children with ASD. This suggests a connection between the genetics of VFGM and immunological abnormalities in ASD, where potentially higher levels of IgA-responsive bacteria may relate to the inflammation observed in individuals with ASD; and (2) VFGM genes were able to distinguish between ASD and TD children, suggesting a novel method for early detection and intervention for ASD, which was supported by machine learning results.

This study has the value of showing a new connection between gut microbiota and the immune system in ASD, where alterations in both have been reported in these individuals. It also introduces VFGM genes into the realm of genetic investigations in ASD and as a parameter for machine learning-based prediction of ASD. Given the increasing importance given to gut-brain axis in pathophysiology of neurological conditions, VFGM genes may become the next hot genetic topic in ASD, as candidate genes and epigenetic signatures are yet to reveal promising diagnostic and therapeutic results.

## 6 Ethics approval and consent to participate

The study was approved by the Ethics Committee of Children’s Hospital of Fudan University.

## 7 Availability of data and materials

Raw data was available at European Nucleotide Archive via ERP104786; the datasets generated and analyzed in this study are available from the corresponding author on reasonable request.

## Funding

This project was supported by National Natural Science Foundation of China (Program Nos. 82071733, 81960290 and 81701351), by Shanghai Municipal Science and Technology Major Project (No.2018SHZDZX01) and ZJLab, by Shanghai talent development funding (No.2020115), by Shenzhen Science Technology and Innovation Commission (JCYJ20190807152403624 and JCYJ20170413093358429), by High Level Project of Medicine in Longhua, ShenZhen (No. HLPM201907020103), and by Longgang Science Technology and Innovation Commission of Shenzhen (LGKCYLWS2018000048).

## Authors contributions

All authors designed and executed the study and wrote the manuscript. All authors read and approved the final manuscript.

## Declaration of Competing Interest

The authors declare that they have no known competing financial interests or personal relationships that could have appeared to influence the work reported in this paper.

## References

[1] Maenner M.J. (2020). Prevalence of autism spectrum disorder among children aged 8 years—autism and developmental disabilities monitoring network, 11 sites, United States, 2016. MMWR Surveill Summ.

[2] Zhou H., Xu X., Yan W., Zou X., Wu L., Luo X. (2020). Prevalence of autism spectrum disorder in China: a nationwide multi-center population-based study among children aged 6 to 12 years. Neurosci Bull.

[3] Alvarez-Mora MI, Escalona RC, Navarro OP, Madrigal I, Quintela I, Amigo J, Martinez-Elurbe D, Linder-Lucht M, Lain GA, Carracedo A, Mila M .Comprehensive molecular testing in patients with high functioning autism spectrum disorder. Mutat Res-Fund Mol M 2016;784:46-52. 10.1016/j.mrfmmm.2015.12.006 26845707

[4] Wang Y., Kasper L.H. (2014). The role of microbiome in central nervous system disorders. Brain Behav Immun.

[5] Vuong H.E., Hsiao E.Y. (2017). Emerging roles for the gut microbiome in autism spectrum disorder. Biol Psychiatry.

[6] Kang D.W. (2017). Microbiota Transfer Therapy alters gut ecosystem and improves gastrointestinal and autism symptoms: an open-label study. Microbiome.

[7] Liu B, Zheng D, Jin Q, Chen L, Yang J. VFDB 2019: a comparative pathogenomic platform with an interactive web interface. Nucleic Acids Research. 2019;47(D1):D687-92 10.1093/nar/gky1080 PMC6324032 30395255

[8] Zhou J., He F., Yang F., Yang Z., Xie Y., Zhou S., Liang J., Xu R., Wang Y., Guo H., Zhou W., Wang M. (2018). Increased stool immunoglobulin A level in children with autism spectrum disorders. Res Dev Disabil.

[9] Mathias A., Pais B., Favre L., Benyacoub J., Corthésy B. (2014). Role of secretory IgA in the mucosal sensing of commensal bacteria. Gut Microbes.

[10] Kawamoto S et al. The inhibitory receptor PD-1 regulates IgA selection and bacterial composition in the gut. Science 2012; 336(6080):485-9 10.1126/science.1217718 22539724

[11] Gutzeit C, Magri G, Cerutti A. Intestinal IgA production and its role in host-microbe interaction. Immunol Rev 2014; 260(1):76-85 10.1111/imr.12189 PMC4174397 24942683

[12] Stahl D., Pickles A., Elsabbagh M., Johnson M.H., The BASIS Team (2012). Novel machine learning methods for ERP Analysis: a validation from research on infants at risk for Autism. Develop Neuropsychol.

[13] Maenner MJ, Yeargin-Allsopp M, Van Naarden Braun K, Christensen DL, Schieve LA. Development of a machine learning algorithm for the surveillance of autism spectrum disorder. PloS one. 2016;11(12):e0168224. 10.1371/journal.pone.0168224 PMC5176307 28002438

[14] Jamal W., Das S., Oprescu I.-A., Maharatna K., Apicella F., Sicca F. (2014). Classification of autism spectrum disorder using supervised learning of brain connectivity measures extracted from synchrostates. J Neural Eng.

[15] Castelhano J., Tavares P., Mouga S., Oliveira G., Castelo-Branco M. (2018). Stimulus dependent neural oscillatory patterns show reliable statistical identification of autism spectrum disorder in a face perceptual decision task. Clin Neurophysiol.

[16] Vicnesh J., Wei J.K., Oh S.L., Arunkumar N., Abdulhay E.W., Ciaccio E.J. (2020). Autism spectrum disorder diagnostic system using HOS bispectrum with EEG signals. Int J Environ Res Public Health.

[17] Shahraki M.F., Farhadyar K., Kavousi K., Azarabad M.H., Boroomand A., Ariaeenejad S. (2020:). A generalized machine-learning aided method for targeted identification of industrial enzymes from metagenome: A xylanase temperature dependence case study. BioRxiv.

[18] Huang L., Xu C., Yang W., Yu R. (2020). A machine learning framework to determine geolocations from metagenomic profiling. Biol Direct.

[19] Lai W.T., Deng W.F., Xu S.X., Zhao J., Xu D., Liu Y.H. (2019). Shotgun metagenomics reveals both taxonomic and tryptophan pathway differences of gut microbiota in major depressive disorder patients. Psychol Med.

[20] Wu T., Wang H., Lu W., Zhai Q., Zhang Q., Yuan W. (2020). Potential of gut microbiome for detection of autism spectrum disorder. Microb Pathog.

[21] Agany D.D.M., Pietri J.E., Gnimpieba E.Z. (2020). Assessment of vector-host-pathogen relationships using data mining and machine learning. Comput Struct Biotechnol J.

[24] Wang M, Wan J, Rong H, He F, Wang H, Zhou J, Cai C, Wang Y, Xu R, Yin Z, Zhou W. Alterations in gut glutamate metabolism associated with changes in gut microbiota composition in children with autism spectrum disorder. MSystems. 2019;4(1). 10.1128/mSystems.00321-18 PMC6351726 30701194

[25] Schopler E., Reichler R.J., DeVellis R.F., Daly K. (1980). Toward objective classification of childhood autism: Childhood Autism Rating Scale (CARS). J Autism Dev Disord.

[26] Wang M., Zhou J., He F., Cai C., Wang H., Wang Y., Lin Y., Rong H., Cheng G., Xu R., Zhou W. (2019). Alteration of gut microbiota-associated epitopes in children with autism spectrum disorders. Brain Behav Immun.

[27] Xu R., Wu B., Liang J., He F., Gu W., Li K., Luo Y.i., Chen J., Gao Y., Wu Z.e., Wang Y., Zhou W., Wang M. (2020). Altered gut microbiota and mucosal immunity in patients with schizophrenia. Brain Behav Immun.

[28] Zhou S., Wang Z., He F., Qiu H., Wang Y., Wang H., Zhou J., Zhou J., Cheng G., Zhou W., Xu R., Wang M. (2019). Association of serum bilirubin in newborns affected by jaundice with gut microbiota dysbiosis. J Nutr Biochem.

[29] Chen L. (2004). VFDB: a reference database for bacterial virulence factors. Nucleic Acids Res.

[30] Buchfink B., Xie C., Huson D.H. (2015). Fast and sensitive protein alignment using DIAMOND. Nat Methods.

[31] Kovtun A.S., Averina O.V., Alekseeva M.G., Danilenko V.N. (2020). Antibiotic resistance genes in the gut microbiota of children with autistic spectrum disorder as possible predictors of the disease. Microb Drug Resist.

[32] Sayers S, Li L, Ong E, Deng S, Fu G, Lin Y, Yang B, Zhang S, Fa Z, Zhao B, Xiang Z. Victors: a web-based knowledge base of virulence factors in human and animal pathogens. Nucleic Acids Res 2019;47(D1):D693-700. 10.1093/nar/gky999 PMC6324020 30365026

[33] Bergeron J.D.L., Deslauriers J., Grignon S., Fortier L.C., Lepage M., Stroh T., Poyart C., Sébire G. (2013). White matter injury and autistic-like behavior predominantly affecting male rat offspring exposed to group b streptococcal maternal inflammation. Dev Neurosci.

[34] Allard M.-J., Bergeron J.D., Baharnoori M., Srivastava L.K., Fortier L.-C., Poyart C., Sébire G. (2017). A sexually dichotomous, autistic-like phenotype is induced by Group B Streptococcus maternofetal immune activation : Group B Streptococcus and autism. Autism Res.

[35] Kirsten TB et al. Lipopolysaccharide exposure induces maternal hypozincemia, and prenatal zinc treatment prevents autistic-like behaviors and disturbances in the striatal dopaminergic and mTOR systems of offspring. PLoS One 2015;10(7):e0134565. 10.1371/journal.pone.0134565 PMC4517817 26218250

[36] Kirsten T.B., Bernardi M.M. (2017). Prenatal lipopolysaccharide induces hypothalamic dopaminergic hypoactivity and autistic-like behaviors: Repetitive self-grooming and stereotypies. Behav Brain Res.

[37] Fernández de Cossío L., Guzmán A., van der Veldt S., Luheshi G.N. (2017). Prenatal infection leads to ASD-like behavior and altered synaptic pruning in the mouse offspring. Brain Behav Immun.

[38] Rolhion N. (2016). Inhibition of nuclear transport of NF-kB p65 by the salmonella type III secretion system effector SpvD. PLoS Pathog.

[39] Haas W., Shepard B.D., Gilmore M.S. (2002). Two-component regulator of Enterococcus faecalis cytolysin responds to quorum-sensing autoinduction. Nature.

[40] Khilwani B, Mukhopadhaya A, Chattopadhyay K. Transmembrane oligomeric form of Vibrio cholerae cytolysin triggers TLR2/TLR6–dependent proinflammatory responses in monocytes and macrophages. Biochem J 2015;466(1):147-61. 10.1042/BJ20140718 25431887

[41] Coburn P.S. (2004). Enterococcus faecalis senses target cells and in response expresses cytolysin. Science.

[42] Kang V., Wagner G.C., Ming X. (2014). Gastrointestinal dysfunction in children with autism spectrum disorders: gastrointestinal dysfunction in autism. Autism Res.

[44] Palm NW et al. Immunoglobulin A coating identifies colitogenic bacteria in inflammatory bowel disease. Cell 2014; 158(5):1000-1010. 10.1016/j.cell.2014.08.006 PMC4174347 25171403

[45] Viladomiu M. (2017). IgA-coated E. coli enriched in Crohn's disease spondyloarthritis promote TH17-dependent inflammation. Sci Transl Med.

[46] Okai S., Usui F., Yokota S., Hori-i Y., Hasegawa M., Nakamura T., Kurosawa M., Okada S., Yamamoto K., Nishiyama E., Mori H., Yamada T., Kurokawa K., Matsumoto S., Nanno M., Naito T., Watanabe Y., Kato T., Miyauchi E., Ohno H., Shinkura R. (2016). High-affinity monoclonal IgA regulates gut microbiota and prevents colitis in mice. Nat Microbiol.

[47] Ege M.J., Mayer M., Normand A.-C., Genuneit J., Cookson W.O.C.M., Braun-Fahrländer C., Heederik D., Piarroux R., von Mutius E. (2011). Exposure to environmental microorganisms and childhood asthma. N Engl J Med.

[48] Edmiston E., Ashwood P., Van de Water J. (2017). Autoimmunity, autoantibodies, and autism spectrum disorder. Biol Psychiatry.

[49] Mathis D., Benoist C. (2011). Microbiota and autoimmune disease: the hosted self. Cell Host Microbe.

[50] Doenyas C. (2018). Gut microbiota, inflammation, and probiotics on neural development in autism spectrum disorder. Neuroscience.

[51] Sharon G., Cruz N.J., Kang D.-W., Gandal M.J., Wang B.o., Kim Y.-M., Zink E.M., Casey C.P., Taylor B.C., Lane C.J., Bramer L.M., Isern N.G., Hoyt D.W., Noecker C., Sweredoski M.J., Moradian A., Borenstein E., Jansson J.K., Knight R., Metz T.O., Lois C., Geschwind D.H., Krajmalnik-Brown R., Mazmanian S.K. (2019). Human gut microbiota from autism spectrum disorder promote behavioral symptoms in mice. Cell.

[52] Wang X., Yang J., Zhang H., Yu J., Yao Z. (2019). Oral probiotic administration during pregnancy prevents autism‐related behaviors in offspring induced by maternal immune activation via anti‐inflammation in mice. Autism Res.

