# Supplementary material for: Virulence factor-related gut microbiota genes and immunoglobulin A levels as novel markers for machine learning-based classification of autism spectrum disorder
Source: Comput Struct Biotechnol J. 2020 Dec 29;19:545–54. doi: 10.1016/j.csbj.2020.12.012 (PMC7809157; doi:10.1016/j.csbj.2020.12.012)
Supplement: Supplementary data 1 [file mmc1.docx]

**Supplementary Appendix**

**Supplementary Figures**


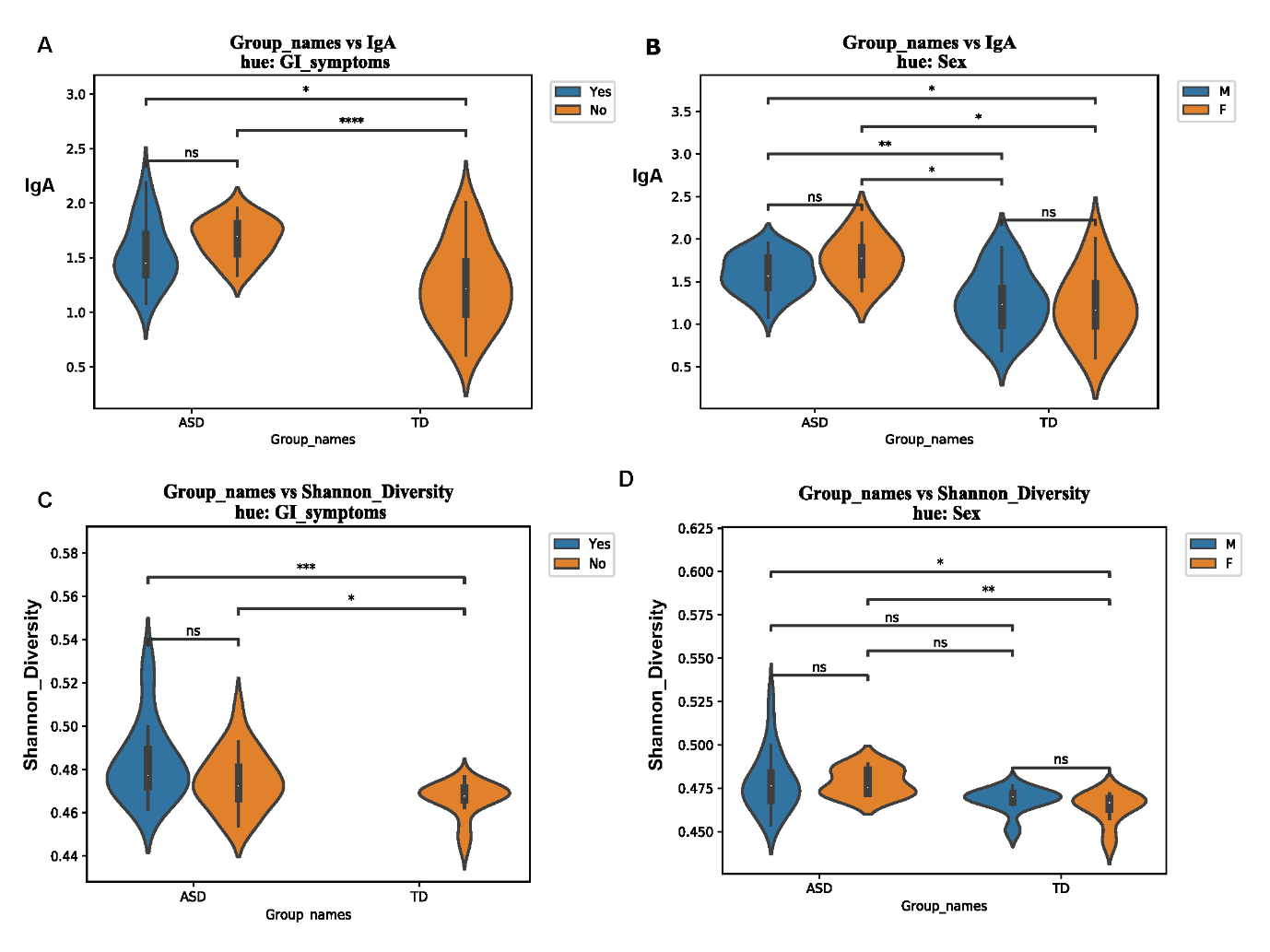


**Supplementary Figure 1. violinplot of GI symptoms and sex's impact on gut IgA level and VFGMs gene diversity.** a, GI symptoms and gut IgA level; b, sex and gut IgA level; c, GI symptoms and VFGMs gene diversity; d, sex and VFGMs gene diversity .

**Supplementary Tables**

Supplementary Table 1. PERMANOVA test of the association between features and VFGM gene composition

Supplementary Table 2. Identification of ASD-enriched or -depleted VFGM genes
